# Serogroup Distribution of *Leptospira* Among Humans and Rodents in Zakarpattia Oblast, Ukraine (2018–2023)

**DOI:** 10.3390/microorganisms13030614

**Published:** 2025-03-07

**Authors:** Pavlo Petakh, Oleksandr Kamyshnyi

**Affiliations:** 1 Department of Biochemistry and Pharmacology, Uzhhorod National University, 88000 Uzhhorod, Ukraine; 2 Department of Microbiology, Virology, and Immunology, I. Horbachevsky Ternopil National Medical University, 46001 Ternopil, Ukraine; kamyshnyi_om@tdmu.edu.ua

**Keywords:** zoonotic infection, public health surveillance, reservoir hosts, microbial epidemiology, disease transmission

## Abstract

Leptospirosis is a zoonotic disease caused by *Leptospira* bacteria, which affects both humans and animals. This study investigated the prevalence of *Leptospira* serogroups in human and rodent reservoirs across Zakarpattia Oblast, Ukraine, from 2018 to 2023. The objective was to identify epidemiological patterns and assess potential public health risks. Data were sourced from the Public Health Center of Ukraine and regional surveillance initiatives, encompassing reported human cases and rodent-carrier detection. Six main serogroups—Icterohaemorrhagiae, Pomona, Grippotyphosa, Hebdomadis, Canicola, and Tarassovi—were included in the analysis. The results showed that Pomona and Hebdomadis serogroups became more common in 2023. The data from different districts also showed regional differences, with Icterohaemorrhagiae and Pomona being the most frequent serogroups in both humans and rodents. Other serogroups, like Grippotyphosa and Canicola, were found less often. Rodents may be an important source of leptospirosis in Zakarpattia. The growing number of cases in recent years shows the need for improved monitoring, control, and prevention in the region.

## 1. Introduction

Leptospirosis is the most widespread zoonotic disease globally, occurring on all continents except Antarctica [1]. It causes over 1 million cases annually, with 60,000 of those being fatal [2]. The incidence rate ranges from 0.1 to 1 per 100,000 per year in temperate cli-mates, from 10 to 100 per 100,000 per year in the humid tropics, and exceeds 100 per 100,000 per year during outbreaks and among high-exposure risk groups [3]. The disease is endemic in regions such as the Caribbean, Central and South America, Southeast Asia, and Oceania [4].

Leptospirosis is caused by bacteria belonging to the order *Spirochaetales*, family *Leptospiraceae*, and genus *Leptospira* [5]. These bacteria are genetically classified into four groups: P1 (pathogenic), P2 (intermediate), and S1 and S2 (saprophytic) [6,7]. They are also serologically categorized based on their serogroups and serovars, which are determined by the antigenic heterogeneity of exposed lipopolysaccharides (LPSs) [8]. Pathogenic leptospires have been classified into more than 24 serogroups and 300 serovars. Although the serovar classification holds epidemiological value, it lacks taxonomic standing [9].

Numerous animals, primarily mammals, serve as reservoirs of *Leptospira* bacteria [10]. Rodents are the most significant and widely distributed reservoir [11]. Specific serovars are associated with particular species of natural maintenance hosts [12,13]. In chronic infections, *Leptospira* bacteria localize in the kidneys, often without causing detectable clinical symptoms [14].

The usual mode of transmission is through abrasions or cuts in the skin or via the conjunctiva, either by direct or indirect contact with the urine or tissues of infected animals [3,15]. Other modes, such as inhalation of water or aerosols, animal bites, or human-to-human transmission, are rare [16].

Leptospirosis is an occupational disease for veterinarians, farmers, abattoir workers, butchers, hunters, and rodent control workers [17]. Indirect contact with contaminated wet soil or water is the primary source of infection in tropical regions, often linked to occupational exposure, such as rice or taro farming, flooding after heavy rains, or exposure during recreational activities [18,19]. Adventure tourism in tropical endemic areas has also contributed to an increase in cases [20,21].

Ukraine has faced significant challenges in recent years that have affected the clinical course and spread of leptospirosis, including the ongoing war and the destruction of the Kakhovka Dam on 6 June 2023 [22]. Military personnel are particularly at risk due to their activities, which often involve prolonged exposure to water and mud (e.g., trenches, dugouts, and shelters), conditions conducive to the transmission of *Leptospira* [23]. A 2022 study by Ogorodniychuk et al. reported that six contract service members and 40 mobilized personnel contracted leptospirosis, accounting for 32% of all cases during the study period [24].

Civilians are also at risk. Potential contact with rodents in poorly maintained bomb shelters can lead to infection. One case report describes a 70-year-old Ukrainian man who contracted leptospirosis. During the Russo–Ukrainian War, he left his house only during air alarms and stayed in the bomb shelter located in the basement of his apartment complex [25]. The authors attributed his infection to unsuitable living conditions in the bomb shelter and direct contact with dried rodent excrement.

Regarding the Kakhovka Reservoir’s destruction, the WHO has expressed concern about potential outbreaks of rodent-borne diseases, including leptospirosis and tularemia [26,27]. Additionally, stress due to war and migration can impair humoral and cellular immune responses to pathogens, increasing susceptibility to infectious diseases, including leptospirosis [28,29].

A preliminary analysis of leptospirosis incidence in Ukraine shows the following annual distribution of cases: 273 cases (0.64 per 100,000) in 2018, 295 cases (0.70 per 100,000) in 2019, 120 cases (0.29 per 100,000) in 2020, 122 cases (0.29 per 100,000) in 2021, 141 cases (0.34 per 100,000) in 2022, and 433 cases (1.056 per 100,000) in 2023 [22]. Particular attention is being given to Zakarpattia Oblast, located on Ukraine’s western border with Romania, Hungary, Poland, and Slovakia, which has an extremely high incidence rate of 12.08 per 100,000 people [30,31].

The aim of this brief report was to investigate the distribution of *Leptospira* serogroups among animals and residents of Zakarpattia to understand the potential causes of the increased incidence of leptospirosis in this region.

## 2. Materials and Methods

The data for this study were collected based on official requests made to the Public Health Center in Kyiv and the Zakarpattia Center for Disease Control and Prevention (Supplementary File S1).

Serogroup identification was carried out in the Especially Dangerous Infections (EDIs) Laboratory at the Zakarpattia Center for Disease Control and Prevention using the microscopic agglutination test (MAT). Initially, paired blood samples were tested at dilutions of 1:5 and 1:50. If a positive reaction occurred at these levels, further testing was done with dilutions of 1:10, 1:100, 1:200, and higher. This process followed the guidelines provided in the Methodological Recommendations 9.1.1.098-02 for Anti-Epidemic Measures and Laboratory Diagnostics of Leptospirosis, approved by the Chief State Sanitary Doctor of Ukraine on 11 December 2002 (Decree No. 39) [32].

To confirm the results, a control culture diluted 1:2 in phosphate-buffered saline was used. The endpoint was determined as the serum dilution at which 50% of the cells agglutinated, leaving 50% free cells, in comparison with the control culture. This method is described in the Leptospirosis Terrestrial Manual by the World Organisation for Animal Health (WOAH) [33]. Results were reported as endpoint serum dilutions or titers, calculated as the reciprocal of the endpoint dilution. Antibody titers of ≥1:100, combined with clinical and epidemiological data, were considered evidence of infection. A fourfold rise in antibody titers between paired samples was strong proof of acute infection. The MAT test was conducted using 14 *Leptospira* serovars (Supplementary File S1), following the protocols outlined locally.

Small mammals were sampled as part of active surveillance following human cases and as part of ongoing monitoring of wild animal infection sources in areas previously identified as leptospirosis hotspots near human settlements [34]. Surveys were conducted across diverse habitats such as farmland, forests, shrubs, grasslands, and areas close to population centers. These habitats represented various geographical zones in the Zakarpattia region, including mountainous, foothill, and lowland areas.

Wild mammals were captured using small snap traps for smaller animals like mice and larger traps for rats. If animals were found alive in the traps, euthanasia was carried out in compliance with national bioethics standards. Captured animals were sent to the EDI laboratory for testing, where blood samples were analyzed using the MAT method, similar to human testing. However, only one blood sample was taken from each animal. During necropsy, blood was collected from the heart cavity onto 1 × 1 cm pieces of filter paper to create “dry blood drops” [35]. These dried samples were later extracted in 0.5 mL of physiological solution (1:10 dilution) and prepared for further testing.

On average, 100–120 surveys were conducted annually by the EDI laboratory staff. During each survey, 80–100 traps were placed approximately every five meters along a transect. The traps were checked, animals collected, and the traps reset one to two times overnight for each survey. Annually, approximately 1500–1800 trap-days were recorded. Historically, epizootological surveys were carried out in each district (rayon) twice a year—once in the spring and again during the summer–autumn period (June to October). The number of traps and their placement in each habitat depended on the district size and the species of animal expected to be captured, as determined by EDI laboratory biologists.

## 3. Results

### 3.1. Human Data

According to the statistical data provided by the Public Health Center, all identified *Leptospira* serogroups were classified into six main categories: Icterohaemorrhagiae, Pomona, Grippotyphosa, Hebdomadis, Canicola, and Tarassovi as well as an “Others” category which included serogroups that did not fall under the mandatory reporting requirements of the Public Health Center of Ukraine. These serogroups, among the 14 studied (as listed in Supplementary File S1), were grouped together in accordance with national regulatory standards.

The annual distribution of cases demonstrated significant variation between serogroups and years.

Cases caused by the Icterohaemorrhagiae serogroup remained relatively stable, with peaks observed in 2018 and 2023, both recording 11 cases. Pomona exhibited a marked increase in 2023, with 29 cases compared to fewer than 7 cases in the preceding years. Similarly, the Hebdomadis serogroup showed a significant rise in 2023, reaching 28 cases, following a period of low detection between 2018 and 2022.

Fluctuations were also observed in other serogroups. Grippotyphosa cases peaked in 2021 with 10 reported cases but dropped to only 1 case in 2023. Canicola, while consistently rare, showed a slight increase in 2023, with five cases reported. The Tarassovi serogroup was not detected throughout the six-year study period. Cases categorized as Others rose dramatically in 2023, with 64 cases reported compared to no more than 4 cases annually in previous years (Table 1).

Overall, the total number of leptospirosis cases saw a significant increase in 2023, driven primarily by the sharp rise in cases attributed to Pomona, Hebdomadis, and Others.

### 3.2. Rodent Data

The results of rodent testing from 2018 to 2023 reflected the detection of *Leptospira* serogroups in small mammal populations. Across the study period, six serogroups were identified: Icterohaemorrhagiae, Pomona, Grippotyphosa, Hebdomadis, Canicola, and Tarassovi, as well as serogroups in the Others category.

The Pomona serogroup showed a marked increase in detections in 2023, with 13 positive samples, compared to 5 in 2021 and occasional detections in earlier years. Grippotyphosa was predominantly identified in 2018 and 2019, with six and five positive samples, respectively, but was not detected in subsequent years.

Icterohaemorrhagiae was only identified once, in 2022, while Hebdomadis was detected in a single sample in 2019. Canicola was extremely rare, with one positive detection in 2023, and Tarassovi was not detected in any year of the study (Table 2).

Samples classified under Others were consistently detected in small numbers throughout the study period, with three detections annually in 2018 and 2019 and two detections in 2023.

### 3.3. Trends Across Districts

The distribution of *Leptospira* serogroups across districts in Zakarpattia Oblast from 2018 to 2023 showed notable variations in the prevalence of specific serogroups by region and year.

In 2018, the most commonly identified serogroups were Icterohaemorrhagiae and Grippotyphosa, with Icterohaemorrhagiae being detected in Mukachevo, Berehove, Khust, and Rakhiv. Grippotyphosa was detected in Uzhhorod and Tyachiv, while Australis was only found in Rakhiv.

In 2019, Icterohaemorrhagiae remained widespread, being identified in Uzhhorod, Berehove, and Khust. Grippotyphosa was detected in Mukachevo, alongside Batavia and Canicola. Additionally, Khust and Rakhiv recorded a wide range of serogroups, including Pomona, Canicola, Autumnalis, Hebdomadis, and Pomona.

The year 2020 marked an increase in the presence of Hebdomadis and Pomona, particularly in Rakhiv, Tyachiv, Mukachevo, and Berehove. Multiple districts, including Tyachiv and Rakhiv, reported overlapping serogroups such as Hebdomadis, Pomona, and Icterohaemorrhagiae.

In 2021, Grippotyphosa was the predominant serogroup across most districts, being identified in Uzhhorod, Mukachevo, Berehove, and Khust. Hebdomadis was exclusively detected in Tyachiv and Rakhiv.

By 2022, fewer serogroups were identified, with Cynopteri being detected in Mukachevo and Uzhhorod. Pomona and Hebdomadis were recorded in Tyachiv and Rakhiv, respectively. Icterohaemorrhagiae and Autumnalis were found in Berehove, while other districts showed no detections.

**Figure 1:**
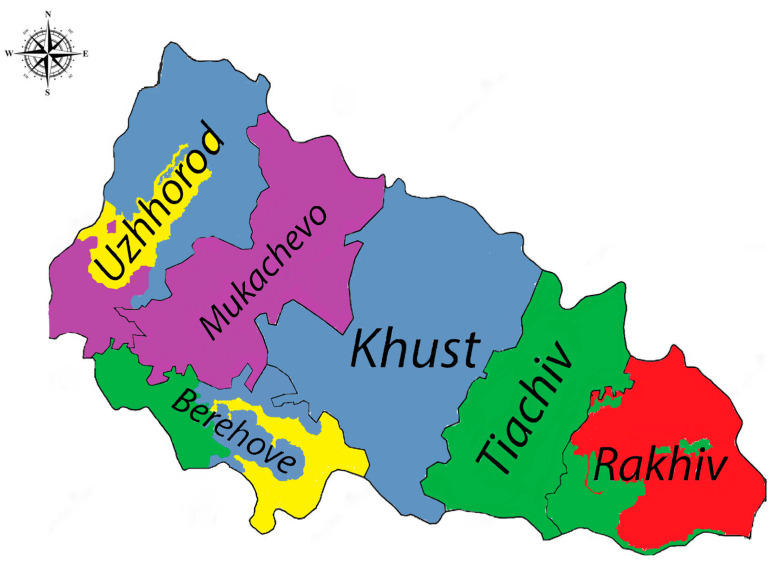
Trends across districts in Zakarpattia Oblast, 2023 (Legend: Sejroe—yellow, Icterohaemorrhagiae—red, Cynopteri—purple, Hebdomadis—grayish blue, Pomona—green). The color distribution in the figure is only relative and represents the prevalence of dominant subgroups across districts.

In 2023, a more diverse range of serogroups was observed. Hebdomadis, Cynopteri, and Sejroe were detected in Uzhhorod, while Cynopteri remained prevalent in Mukachevo. Berehove recorded Hebdomadis, Pomona, and Sejroe, and Pomona was dominant in Tyachiv. Rakhiv reported the presence of Icterohaemorrhagiae and Pomona, indicating a continued risk of rodent-related leptospirosis transmission (Figure 1).

Overall, these findings highlight distinct geographic patterns of *Leptospira* serogroups, with Pomona, Icterohaemorrhagiae, and Hebdomadis emerging as key serogroups in recent years, especially in Rakhiv and Tyachiv.

## 4. Discussion

Leptospirosis is one of the most widespread zoonotic diseases, with particular significance during periods of military conflict. Studies have highlighted its potential impact on military personnel under specific environmental and occupational conditions. For example, Hadad et al. reported an outbreak among Israeli troops following a military exercise near the Jordan River, with *Leptospira* interrogans serovar Hardjo identified as the causative agent [36]. Similarly, outbreaks have been documented in Brazil, where Lupi et al. described cases of leptospirosis in military personnel after field exercises, with symptoms ranging from acute meningoencephalitis to respiratory illness and skin rash. The implicated serovars in this instance included Icterohaemorrhagiae, Hebdomadis, Patoc, and Cynopteri [37]. Furthermore, Burns et al. reported a case of leptospirosis in a soldier returning from Borneo, presenting with fever and acute kidney injury caused by *Leptospira* spp. [38]. These cases emphasize the global importance of leptospirosis in military and occupational settings.

In Ukraine, the number of leptospirosis cases is about 1 per 100,000 people, which matches the local climate and geography [39]. However, Zakarpattia has a much higher rate, with 12.08 cases per 100,000 in 2023. In 2024, the rate dropped to 3.95 per 100,000, with 49 cases reported by the Center for Public Health [22].

Zakarpattia, as the most remote region of Ukraine, shares borders with four EU countries—Romania, Hungary, Poland, and Slovakia—adding complexity to the region’s leptospirosis epidemiology [40]. The region’s low urbanization rate, with over 60% of the population living in rural areas, contributes to higher exposure risks [41]. Many rural households do not have centralized water supplies and use natural water sources, which are often of poor quality [42]. In addition, most people in rural areas work in livestock farming, which increases the risk of zoonotic transmission [43].

Geographical and environmental factors further exacerbate the situation [26]. Zakarpattia has the densest river network in Ukraine, with extensive riparian habitats and surface water bodies, conditions known to correlate with higher leptospirosis incidence [44]. Seasonal flooding, particularly in spring and early summer, creates ideal conditions for rodent intrusion into homes and increased exposure to contaminated water.

Rodents play a significant role in the transmission of leptospirosis in Zakarpattia. According to Markovych et al., the region’s fauna includes approximately 80 mammal species, with over 45% being rodents [34]. In a study conducted between 2005 and 2015, 2820 small mammals were tested, and antibodies to *Leptospira* spp. were detected in 9.79% of the sampled population. The most common species identified as carriers were *Apodemus agrarius*, *Rattus norvegicus*, and *Mus musculus*. The prevalence of *Leptospira* spp. varied among rodent species but was generally between 10 and 15% for well-represented species, except for *Mus musculus*, where prevalence was lower.

The dominant serogroups of *Leptospira* in rodents have shifted over time. From 2005 to 2009, Icterohaemorrhagiae (44.6%) and Pomona (34.4%) were predominant. Between 2010 and 2012, Pomona became the dominant serogroup (50.9%), while from 2013 to 2015, Grippotyphosa accounted for 53.2% of all positive cases. Rare serogroups, such as Australis, Ballum, Bataviae, and Autumnalis, were also found during this time. This shows how the epidemiology of leptospirosis in the region is changing over time.

The findings underscore the need for targeted public health interventions in Zakarpattia, including improved water quality, rodent control measures, and community education on preventive practices. Additionally, enhanced surveillance and monitoring of leptospirosis in both human and animal populations are critical to mitigating the impact of this disease in high-risk areas. In this study, we found that the most important serogroups detected in Zakarpattia were Pomona and Hebdomadis. Each has distinct potential hosts and distribution patterns. Hebdomadis is associated with mice, voles, rats, and cattle, with a geographical distribution primarily in Japan and Europe [45]. On the other hand, the Pomona serogroup is most commonly linked to swine and cattle, especially in the USA and Europe [46]. The identification of these serogroups highlights the role of both domestic and wild animals in maintaining leptospirosis reservoirs in Zakarpattia.

This study had some important limitations. One of the main issues was the possibility of under-reporting of leptospirosis cases, especially in rural areas where people may not have easy access to healthcare or testing. Since the symptoms of leptospirosis can look like other common illnesses, some cases might not have been correctly identified.

The low number of *Leptospira* detections in rodents from 2020 to 2022 might be because of the COVID-19 pandemic. Restrictions during the pandemic, like travel bans and lockdowns, made it harder to collect samples and conduct field studies. Also, laboratories were focused on responding to COVID-19, which may have delayed or reduced testing for leptospirosis.

Another limitation was that this study only looked at a small number of rodent species and *Leptospira* serovars. This means it may not fully represent all the possible animals and serogroups spreading leptospirosis in the region.

Finally, environmental changes, like seasonal flooding and climate differences, might have affected the results. The study did not focus on these factors, even though they can influence the spread of *Leptospira* in both rodents and humans.

## Figures and Tables

**Table 1:** Distribution of *Leptospira* serogroups in humans in Zakarpattia Oblast (2018–2023).

Year	2018	2019	2020	2021	2022	2023
Icterohaemorrhagiae	11	5	2	2	2	11
Pomona	2	2	7	0	1	29
Grippotyphosa	5	1	4	10	0	1
Hebdomadis	1	5	7	3	0	28
Canicola	2	2	1	0	0	5
Tarassovi	0	0	0	0	0	0
Others	3	4	1	2	4	64

**Table 2:** Detection of *Leptospira* serogroups in rodents in Zakarpattia Oblast (2018–2023).

Year	2018	2019	2020	2021	2022	2023
Icterohaemorrhagiae	0	0	0	0	1	0
Pomona	1	2	0	5	0	13
Grippotyphosa	6	5	0	1	0	0
Hebdomadis	0	1	0	0	0	0
Canicola	0	0	0	0	0	1
Tarassovi	0	0	0	0	0	0
Others	3	3	0	1	0	2

## Data Availability

The original contributions presented in this study are included in the article and Supplementary Materials. Further inquiries can be directed to the corresponding author.

## Supplementary Materials

The following supporting information can be downloaded at: https://www.mdpi.com/article/10.3390/microorganisms13030614/s1, Supplementary File S1: Official Response on Leptospirosis Incidence and Serogroup Prevalence in Ukraine and Zakarpattia oblast. Supplementary File S2: Incidence of leptospirosis by district in Zakarpattia oblast in 2023.

## References

[1] Adler B., de la Peña Moctezuma A. (2010). Leptospira and leptospirosis. J. Vet. Microbiol..

[2] Costa F., Hagan J.E., Calcagno J., Kane M., Torgerson P., Martinez-Silveira M.S., Stein C., Abela-Ridder B., Ko A.I. (2015). Global morbidity and mortality of leptospirosis: A systematic review. PLoS Neglected Trop. Dis..

[3] Musso D., La Scola B. (2013). Laboratory diagnosis of leptospirosis: A challenge. J. Microbiol. Immunol. Infect..

[4] Pappas G., Papadimitriou P., Siozopoulou V., Christou L., Akritidis N. (2008). The globalization of leptospirosis: Worldwide incidence trends. Int. J. Infect. Dis. IJID Off. Publ. Int. Soc. Infect. Dis..

[5] Rahman M.M., Islam M.R., Dhar P.S. (2023). Leptospirosis’s abrupt resurgence: Types, bacteriology, molecular genetics, etiology, diagnostic testing, transmission, symptoms, and medications. Int. J. Surg..

[6] Vanithamani S., Akino Mercy C.S., Kanagavel M., Sumaiya K., Bothammal P., Saranya P., Prasad M., Ponmurugan K., Muralitharan G., Al-Dhabi N.A. (2021). Biochemical analysis of leptospiral LPS explained the difference between pathogenic and non-pathogenic serogroups. Microb. Pathog..

[7] Pětrošová H., Mikhael A., Culos S., Giraud-Gatineau A., Gomez A.M., Sherman M.E., Ernst R.K., Cameron C.E., Picardeau M., Goodlett D.R. (2023). Lipid A structural diversity among members of the genus Leptospira. Front. Microbiol..

[8] Ko A.I., Goarant C., Picardeau M. (2009). Leptospira: The dawn of the molecular genetics era for an emerging zoonotic pathogen. Nat. Rev. Microbiol..

[9] Evangelista K.V., Coburn J. (2010). Leptospira as an emerging pathogen: A review of its biology, pathogenesis and host immune responses. Future Microbiol..

[10] Medeiros L.d.S., Braga Domingos S.C., Azevedo M.I.N.D., Peruquetti R.C., de Albuquerque N.F., D’Andrea P.S., Botelho A.L.d.M., Crisóstomo C.F., Vieira A.S., Martins G. (2020). Small Mammals as Carriers/Hosts of *Leptospira* spp. in the Western Amazon Forest. Front. Vet. Sci..

[11] Cosson J.F., Picardeau M., Mielcarek M., Tatard C., Chaval Y., Suputtamongkol Y., Buchy P., Jittapalapong S., Herbreteau V., Morand S. (2014). Epidemiology of leptospira transmitted by rodents in southeast Asia. PLoS Negl. Trop. Dis..

[12] Hathaway S.C., Blackmore D.K., Marshall R.B. (1983). Leptospirosis and the maintenance host: A laboratory mouse model. Res. Vet. Sci..

[13] Cilia G., Bertelloni F., Albini S., Fratini F. (2021). Insight into the Epidemiology of Leptospirosis: A Review of Leptospira Isolations from “Unconventional” Hosts. Animals.

[14] Chou L.F., Yang H.Y., Hung C.C., Tian Y.C., Hsu S.H., Yang C.W. (2023). Leptospirosis kidney disease: Evolution from acute to chronic kidney disease. Biomed. J..

[15] Bradley E.A., Lockaby G. (2023). Leptospirosis and the Environment: A Review and Future Directions. Pathogens.

[16] Rahman M.T., Sobur M.A., Islam M.S., Ievy S., Hossain M.J., El Zowalaty M.E., Rahman A.T., Ashour H.M. (2020). Zoonotic Diseases: Etiology, Impact, and Control. Microorganisms.

[17] Goarant C. (2016). Leptospirosis: Risk factors and management challenges in developing countries. Res. Rep. Trop. Med..

[18] Brito Monteiro M., Egídio de Sousa I., Piteira M., Coelho S., Freitas P. (2021). Leptospirosis, a Re-emerging Threat. Cureus.

[19] Wynwood S.J., Graham G.C., Weier S.L., Collet T.A., McKay D.B., Craig S.B. (2014). Leptospirosis from water sources. Pathog. Glob. Health.

[20] Trubo R. (2001). Leptospira brings fresh challenge to adventure sports. Lancet Infect. Dis..

[21] Lau C., Smythe L., Weinstein P. (2010). Leptospirosis: An emerging disease in travellers. Travel. Med. Infect. Dis..

[22] Petakh P., Tymchyk V., Kamyshnyi O. (2024). Surveillance of human leptospirosis infections in Ukraine between 2018 and 2023. Front. Public Health.

[23] Petakh P., Oksenych V., Kamyshna I., Boisak I., Lyubomirskaya K., Kamyshnyi O. (2024). Exploring the complex interplay: Gut microbiome, stress, and leptospirosis. Front. Microbiol..

[24] Ogorodniychuk I., Soroka N., Ovcharuk V., Ovcharuk N. (2023). Epidemiologic features of leptospirosis among the population of Ukraine and in military collectives. Ukr. J. Mil. Med..

[25] Zubach O., Pestushko I., Dliaboha Y., Semenyshyn O., Zinchuk A. (2023). A Single Clinical Case of Leptospirosis in a 70-Year-Old Man During the Military Conflict in Ukraine. Vector Borne Zoonotic Dis..

[26] Petakh P., Huber W., Kamyshnyi O. (2024). Geographical factors and air raid alarms influence leptospirosis epidemiology in Ukraine (2018–2023). One Health.

[27] WHO Steps up Its Humanitarian Response in Southern Ukraine Following the Destruction of the Kakhovka Dam. https://reliefweb.int/report/ukraine/who-steps-its-humanitarian-response-southern-ukraine-following-destruction-kakhovka-dam.

[28] Petakh P., Oksenych V., Kamyshna I., Boisak I., Lyubomirskaya K., Kamyshnyi O. (2024). Exploring the interplay between posttraumatic stress disorder, gut microbiota, and inflammatory biomarkers: A comprehensive meta-analysis. Front. Immunol..

[29] Seiler A., Fagundes C.P., Christian L.M., Choukèr A. (2020). The Impact of Everyday Stressors on the Immune System and Health. Stress Challenges and Immunity in Space: From Mechanisms to Monitoring and Preventive Strategies.

[30] Petakh P., Kamyshnyi A., Tymchyk V., Armitage R. (2023). Infectious diseases during the Russian-Ukrainian war—Morbidity in the Transcarpathian region as a marker of epidemic danger on the EU border. Public Health Pract..

[31] Petakh P., Tymchyk V., Kamyshnyi O. (2024). Communicable diseases in Ukraine during the period of 2018–2023: Impact of the COVID-19 pandemic and war. Travel Med. Infect. Dis..

[32] (2002). Anti-Epidemic Measures and Laboratory Diagnostics of Leptospirosis Approved by the Decree of the Chief State Sanitary Doctor of Ukraine No. 39 of 11 December 2002.

[33] World Organization for Animal Health (WOAH) (2024). Manual of Diagnostic Tests and Vaccines for Terrestrial Animals.

[34] Markovych O., Tymchyk V., Kolesnikova I. (2019). Leptospirosis in Zakarpattia Oblast (2005–2015). Vector-Borne Zoonotic Dis..

[35] Arkell P., Angelina J., do Carmo Vieira A., Wapling J., Marr I., Monteiro M., Matthews A., Amaral S., da Conceicao V., Kim S.H. (2022). Integrated serological surveillance of acute febrile illness in the context of a lymphatic filariasis survey in Timor-Leste: A pilot study using dried blood spots. Trans. R. Soc. Trop. Med. Hyg..

[36] Hadad E., Pirogovsky A., Bartal C., Moran-Gilad J., Barnea A., Yitzhaki S., Grotto I., Balicer R., Schwartz E. (2006). An outbreak of leptospirosis among Israeli troops near the Jordan River. Am. J. Trop. Med. Hyg..

[37] Lupi O., Netto M.A.C., Avelar K., Romero C., Bruniera R., Brasil P. (2013). Cluster of leptospirosis cases among military personnel in Rio de Janeiro, Brazil. Int. J. Infect. Dis..

[38] Burns D.S., Clay K.A., Bailey M.S. (2016). Leptospirosis in a British soldier after travel to Borneo. J. R. Army Med. Corps.

[39] Chadsuthi S., Chalvet-Monfray K., Wiratsudakul A., Modchang C. (2021). The effects of flooding and weather conditions on leptospirosis transmission in Thailand. Sci. Rep..

[40] Petakh P., Kamyshnyi A. (2023). Risks of outbreaks: The health concerns of internally displaced persons in Transcarpathia, Ukraine. New Microbes New Infect..

[41] Hudzelyak I. (2018). Geographical aspects of the demographic situation in Western Ukraine. Visnyk Lviv. Univ. Ser. Geogr..

[42] Nikolaichuk V.V.M., Shpontak J., Karpu’k M. (2015). The current state of water resources of Transcarpathia. Biosyst. Divers..

[43] Zhang T., Nickerson R., Zhang W., Peng X., Shang Y., Zhou Y., Luo Q., Wen G., Cheng Z. (2024). The impacts of animal agriculture on One Health—Bacterial zoonosis, antimicrobial resistance, and beyond. One Health.

[44] Wasiński B., Dutkiewicz J. (2013). Leptospirosis-current risk factors connected with human activity and the environment. Ann. Agric. Environ. Med. AAEM.

[45] Thiermann A.B. (1982). Experimental leptospiral infections in pregnant cattle with organisms of the Hebdomadis serogroup. Am. J. Vet. Res..

[46] Aliberti A., Blanda V., Di Marco Lo Presti V., Macaluso G., Galluzzo P., Bertasio C., Sciacca C., Arcuri F., D’Agostino R., Ippolito D. (2022). Leptospira interrogans Serogroup Pomona in a Dairy Cattle Farm in a Multi-Host Zootechnical System. Vet. Sci..

