# Supplementary material for: Serogroup Distribution of Leptospira Among Humans and Rodents in Zakarpattia Oblast, Ukraine (2018–2023)
Source: Microorganisms. 2025 Mar 7;13(3):614. doi: 10.3390/microorganisms13030614 (PMC11945033; doi:10.3390/microorganisms13030614)
Supplement: Supplementary file 1 [file microorganisms-13-00614-s001.zip › Supplementary File S1.pdf.pdf]

МІНІСТЕРСТВО ОХОРОНИ ЗДОРОВ'Я УКРАЇНИ
ДЕРЖАВНА УСТАНОВА
„ЗАКАРПАТСЬКИЙ ОБЛАСНИЙ ЦЕНТР КОНТРОЛЮ ТА ПРОФІЛАКТИКИ ХВОРОБ МІНІСТЕРСТВА ОХОРОНИ ЗДОРОВ'Я УКРАЇНИ"
м. Ужгород, вул. Собранецька, 96, 88000; тел./факс: 64-28-77,
e-mail: zaklabcentr@zakarpat-ses.gov.ua, zaklabcentr@gmail.com
веб-сайт http://www.zakarpat-ses.gov.ua
кодЄДРПОУ38475462

18.10.2024 № 2142/01 на № ____________ від ____________

Павлу ПЕТАХУ

Відповідь на запит від 14.10.2024 року

ДУ «Закарпатський обласний центр контролю та профілактики хвороб МОЗ України» на Ваш запит від 14.10.2024 р. надає інформацію про поширеність серогруп лептоспір серед населення Закарпатської області в розрізі районів за період 2018-2023 рр. (додаток)

Додаток: таблиця на 2-х аркушах

Генеральний директор **Галина СІТНИК**

Жиган 0501065934
Берташ 0508620033

Додаток

Поширеність серогруп лептоспір серед населення Закарпатської області в розрізі районів за період 2018-2023 рр.

№п/п	Район	2018 рік	2019 рік	2020 рік	2021 рік	2022 рік	2023 рік
1.	**Ужгородський**	При досліджені матеріалу від хворих виявлено антитіла до 6 серогруп лептоспір, ведуче місце – лептоспіра Грипотифоза.	При досліджені матеріалу від хворих виявлено антитіла до 3 серогруп лептоспір, ведуче місце – лептоспіра Іктерогеморагія.	При досліджені матеріалу від хворих виявлено антитіла до 3 серогруп лептоспір, ведуче місце – лептоспіра Гебдомадіс	При досліджені матеріалу від хворих виявлено антитіла до 2 серогруп лептоспір, ведуче місце – лептоспіра Грипотифоза.	Матеріал від хворих на дослідження не доставлявся	При досліджені матеріалу від хворих виявлено антитіла до 7 серогруп лептоспір, переважали в одинаковій кількості – лептоспіра Гебдомадіс, Ціноптері, Сейро
2.	**Мукачівський**	При досліджені матеріалу від хворих виявлено антитіла до 2 серогруп лептоспір, ведуче місце – лептоспіра Іктерогеморагія.	При досліджені матеріалу від хворих виявлено антитіла до 3 серогруп лептоспір, в одинаковій кількості – лептоспіра Грипотифоза, Батавія, Канікола.	При досліджені матеріалу від хворих виявлено антитіла до 3 серогруп лептоспір, ведуче місце – лептоспіра Гебдомадіс та лептоспіра Помона	При досліджені матеріалу від хворих виявлено антитіла до 1 серогрупи лептоспір, лептоспіра Грипотифоза.	При досліджені матеріалу від хворих виявлено антитіла до 2 серогруп лептоспір, ведуче місце – лептоспіра Ціноптері.	При досліджені матеріалу від хворих виявлено антитіла до 7 серогруп лептоспір, ведуче місце – лептоспіра Ціноптері
3.	**Берегівський**	При досліджені матеріалу від хворих виявлено антитіла до 2 серогруп лептоспір, ведуче місце – лептоспіра Іктерогеморагія.	При досліджені матеріалу від хворих виявлено антитіла до 2 серогруп лептоспір, ведуче місце – лептоспіра Іктерогеморагія.	При досліджені матеріалу від хворих виявлено антитіла до 3 серогруп лептоспір, ведуче місце – лептоспіра Помона	При досліджені матеріалу від хворих виявлено антитіла до 3 серогруп лептоспір, ведуче місце – лептоспіра Грипотифоза.	При досліджені матеріалу від хворих виявлено антитіла до 2 серогруп лептоспір, в одинаковій кількості – лептоспіра Іктерогеморагія, Аутумналіс.	При досліджені матеріалу від хворих виявлено антитіла до 3 серогруп лептоспір, в одинаковій кількості – лептоспіра Гебдомадіс, Сейро, Помона.
4.	**Хустський**	При досліджені матеріалу від хворих виявлено антитіла до 2 серогруп лептоспір, ведуче місце – лептоспіра Іктерогеморагія.	При досліджені матеріалу від хворих виявлено антитіла до 5 серогруп лептоспір, в одинаковій кількості – лептоспіра Іктерогеморагія, Помона, Канікола, Аустраліс, Гебдомадіс	При досліджені матеріалу від хворих виявлено антитіла до 1 серогрупи лептоспір, лептоспіра Грипотифоза.	При досліджені матеріалу від хворих виявлено антитіла до 6 серогруп лептоспір, ведуче місце – лептоспіра Грипотифоза.	Матеріал від хворих на дослідження не доставлявся	При досліджені матеріалу від хворих виявлено антитіла до 7 серогруп лептоспір, ведуче місце – лептоспіра Гебдомадіс
5.	**Тячівський**	При досліджені матеріалу від хво-	При досліджені матеріалу від хво-	При досліджені матеріалу від хво-	При досліджені матеріалу від хво-	При досліджені матеріалу від хво-	матеріалу від хворих виявлено анти-

		рих виявлено анти-тіла до 2 серогруп лептоспір, ведуче місце – лептоспіра Грипотифоза.	рих виявлено анти-тіла до 3 серогруп лептоспір, ведуче місце – лептоспіра Гебдомадіс	рих виявлено анти-тіла до 3 серогруп лептоспір, в однаковій кількості – лептоспіра Іктерогеморагія, Помона, Гебдомадіс	рих виявлено анти-тіла до 1 серогрупи лептоспір, лептоспіра Гебдомадіс.	рих виявлено анти-тіла до 1 серогрупи лептоспір, лептоспіра Помона	тіла до 3 серогруп лептоспір, ведуче місце – лептоспіра Помона
6.	**Рахівський**	Матеріал від хворих на дослідження не доставлявся	Матеріал від хворих на дослідження не доставлявся	Матеріал від хворих на дослідження не доставлявся	Матеріал від хворих на дослідження не доставлявся	Матеріал від хворих на дослідження не доставлявся	При досліджені матеріалу від хворих виявлено анти-тіла до 2 серогруп лептоспір, ведуче місце – лептоспіра Помона
7.	**По області**	При досліджені матеріалу від хворих виявлено анти-тіла до 7 серогруп лептоспір, ведуче місце – лептоспіра Іктерогеморагія.	При досліджені матеріалу від хворих виявлено анти-тіла до 7 серогруп лептоспір, ведуче місце – лептоспіра Іктерогеморагія.	При досліджені матеріалу від хворих виявлено анти-тіла до 6 серогруп лептоспір, в однаковій кількості – лептоспіра Гебдомадіс та лептоспіра Помона.	При досліджені матеріалу від хворих виявлено анти-тіла до 5 серогруп лептоспір, ведуче місце – лептоспіра Грипотифоза	При досліджені матеріалу від хворих виявлено анти-тіла до 4 серогруп лептоспір, ведуче місце – лептоспіра Ціноптері	При досліджені матеріалу від хворих виявлено анти-тіла до 10 серогруп лептоспір, ведуче місце – лептоспіра Ціноптері

ДЕРЖАВНА УСТАНОВА
«ЦЕНТР ГРОМАДСЬКОГО ЗДОРОВ’Я
МІНІСТЕРСТВА ОХОРОНИ ЗДОРОВ’Я УКРАЇНИ»
вул. Ярославська, 41, м. Київ, 04071, тел. (044) 334-56-89
E-mail: info@phc.org.ua, код ЄДРПОУ 40524109

______________ № ______________

На № б/н від «14» жовтня 2024 року
На № 26-04/17/3613/ЗПІ-24//3718 від «14» жовтня 2024 року

Павлу Петаху
pavlo.petakh@uzhnu.edu.ua

Міністерство охорони здоров’я України

Державна установа «Центр громадського здоров’я Міністерства охорони здоров’я України» за результатом розгляду запитів про надання публічної інформації Петаха П. від 14.10.2024 № б/н, надісланого листом Міністерства охорони здоров’я України від 14.10.2024 № 26-04/17/3613/ЗПІ-24//3718, в межах компетенції, повідомляє наступне.

Відповідно до пункту 1 розділу І Статуту Державної установи «Центр громадського здоров’я Міністерства охорони здоров’я України», затвердженого наказом Міністерства охорони здоров’я України від 09 лютого 2024 року № 224 (далі - Статут), Центр є санітарно-профілактичним закладом охорони здоров’я, головним завданням якого є діяльність у галузі громадського здоров’я, а саме: здійснення епідеміологічного нагляду, виконання повноважень щодо захисту населення від інфекційних та неінфекційних хвороб, лабораторній діяльності, біологічної безпеки та біологічного захисту у межах, визначених цим Статутом.

В межах компетенції, Центр надає дані щодо етіологічної структури лептоспір в України за 2018-2023 роки, яка збирається згідно галузевої статистичної звітної форми №4 0-здоров «Звіт про роботу санітарно-епідеміологічної (дезінфекційної, протичумної) станції», затвердженої наказом Міністерства охорони здоров’я України від 04.04.2001 року №132 (додається).

Зазначимо, що відповідно до статті 21 Закону України «Про доступ до публічної інформації», у разі, якщо задоволення запиту на інформацію передбачає виготовлення копій документів обсягом більш як 10 сторінок, запитувач зобов'язаний відшкодувати фактичні витрати на копіювання та друк.

Розмір фактичних витрат на виготовлення цифрових копій документів шляхом сканування визначено Центром на підставі розміру фактичних витрат на копіювання або друк документів, що надаються за запитом на інформацію, розпорядником якої є Державна установа «Центр громадського здоров’я України Міністерства охорони здоров’я України», затвердженого наказом Центру від 05.03.2021 № 09-од (у редакції наказу Центру від 22.09.2022 № 39-од), з

№ 04-11/04/760-к/883-к/24 від 17.10.2024
КЕП РОСАДА МИХАЙЛО ОЛЕКСІЙОВИЧ
5E984D526F82F38F04000000182E4F01F83DE404
ДУ "Центр громадського здоров'я МОЗ України"
№ 04-11/04/760-к/883-к/24 від 17.10.2024

урахуванням граничних норм витрат на копіювання або друк документів, що надаються за запитом на інформацію, затверджених постановою Кабінету Міністрів України від 13.07.2011 № 740 (у редакції постанови Кабінету Міністрів України від 15.01.2020 № 4).

З огляду на зазначене вище, Центр надає перші 10 сторінок запитуваної інформації.

Додатково інформуємо, що відповідно до положення статті 21 Закону України «Про доступ до публічної інформації», решту запитуваних документів буде надано після сплати вартості фактичних витрат на копіювання або друк, відповідно до рахунку від «16» жовтня 2024 року № 78 (додається).

Додатки: 1. Рахунок від 16.10.2024 № 78 на 1 арк. в 1 прим;
2. Дані щодо етіологічної структури лептоспір в Україні за 2018р., на 3 арк. в 1 прим;
3. Дані щодо етіологічної структури лептоспір в Україні за 2019р., на 3 арк. в 1 прим;
4. Дані щодо етіологічної структури лептоспір в Україні за 2020р., на 3 арк. в 1 прим;
5. Дані щодо етіологічної структури лептоспір в Україні за 2021р., на 1 арк. в 1 прим.

Генеральний директор **Михайло РОСАДА**

Зінченко Віталій (044) 334-56-89
Полянська Людмила

Додаток 1 до листа
ДУ "Центр громадського здоров'я
МОЗ України"
від ________ №______________

Таблиця 26. Етіологічна структура лептоспір

Хворі і особи з підозрою на захворювання

Область	№ ряд-ка	Всього з позитивним результатом		і з н и х :													
				Іктеро		Помона		Грипотиф.		Гебдомад.		Канікола		Тарасов.		Інші	
		бак.	серол.	бак.	сер.	бак.	сер.	бак.	сер.	бак.	сер.	бак.	сер.	бак.	сер.	бак.	сер.
А	Б	1	2	3	4	5	6	7	8	9	10	11	12	13	14	15	16
АР Крим																	
Вінницька			17		9								3				5
Волинська			3				1		1				1				
Дніпропетров.			1		1												
Донецька																	
Житомирська			1														1
Закарпатська			24		11		2		5		1		2				3
Запорізька																	
Івано-Франк.			28		15		1		4								8
Київська			3		1		1										1
Кіровоград.			11				3		2				2		3		1
Луганська																	
Львівська			21		1		2		1		3		2		1		11
Миколаївська			46		2		1				1						42
Одеська			3		1												2
Полтавська			3		2						1						
Рівненська			1										1				
Сумська			6		3												3
Тернопільська			15		6		1		2		4		2				
Харківська																	
Херсонська			29		5		6		4		2		2		1		9
Хмельницька			21		7		1		2		3		3				5
Черкаська			5		3				2								
Чернівецька			18		5		3		2		2						6
Чернігівська			2		2												
м.Київ			9		4		1		3				1				
м.Севастоп.																	
ЛЦ на ВТ																	
ЛЦ на ЗТ																	
ЛЦ на ПТ																	
**УКРАЇНА**			**267**		**78**		**23**		**28**		**17**		**19**		**5**		**97**

Таблиця 26. Етіологічна структура лептоспір

Особи з профілактичною метою

Область	№ ряд-ка	Всього з позитивним результатом		і з н и х :													
				Іктеро		Помона		Грипотиф.		Гебдомад.		Канікола		Тарасов.		Інші	
		бак.	серол.	бак.	сер.	бак.	сер.	бак.	сер.	бак.	сер.	бак.	сер.	бак.	сер.	бак.	сер.
А	Б	1	2	3	4	5	6	7	8	9	10	11	12	13	14	15	16
АР Крим																	
Вінницька																	
Волинська																	
Дніпропетров.																	
Донецька																	
Житомирська																	
Закарпатська			2		2												

Запорізька																	
Івано-Франк.																	
Київська																	
Кіровоград.																	
Луганська																	
Львівська																	
Миколаївська																	
Одеська																	
Полтавська																	
Рівненська																	
Сумська																	
Тернопільська																	
Харківська																	
Херсонська																	
Хмельницька																	
Черкаська																	
Чернівецька																	
Чернігівська																	
м.Київ																	
м.Севастоп.																	
ЛЦ на ВТ																	
ЛЦ на ЗТ																	
ЛЦ на ПТ																	
СЕС СМСЧ																	
Крим.ПЧС																	
**УКРАЇНА**			2		2												

Таблиця 26. Етіологічна структура лептоспір

Об'єкти довкілля

Область	№ ряд-ка	Всього з позитивним результатом		із них:													
				Іктеро		Помона		Грипотиф.		Гебдомад.		Канікола		Тарасов.		Інші	
		бак.	серол.	бак.	сер.	бак.	сер.	бак.	сер.	бак.	сер.	бак.	сер.	бак.	сер.	бак.	сер.
А	Б	1	2	3	4	5	6	7	8	9	10	11	12	13	14	15	16
АР Крим																	
Вінницька			3								3						
Волинська			79		2		7		18		27		21		3		1
Дніпропетров.			70		25		6		4				10				25
Донецька																	
Житомирська			3		1				1		1						
Закарпатська			10				1		6								3
Запорізька																	
Івано-Франк.			11				2						3				6
Київська			22		5				7				2				8
Кіровоград.			28		2		4		3		4		5		3		7
Луганська																	
Львівська			130		2		12										116
Миколаївська			43		3				9		2		3				26
Одеська																	
Полтавська			26		3						1						22
Рівненська			14						3								11
Сумська			6						1				1		1		3

Тернопільська		20	39		18		4		10		3		4			20	
Харківська			36		8		2		6		20						
Херсонська			50		3		8		2		8				10		19
Хмельницька			18				1		11		4		2				
Черкаська																	
Чернівецька			38		4		12		10		8						4
Чернігівська			41		5		7		1		6		5		2		15
м.Київ			12		5				7								
м.Севастоп.																	
ЛЦ на ВТ																	
ЛЦ на ЗТ																	
ЛЦ на ПТ																	
СЕС СМСЧ																	
Крим.ПЧС																	
**УКРАЇНА**		**20**	**679**		**86**		**66**		**99**		**87**		**56**		**19**	**20**	**266**

Додаток 2 до листа
ДУ "Центр громадського здоров'я
МОЗ України"
від ________ №______________

Таблиця 26. Етіологічна структура лептоспір

Хворі і особи з підозрою на захворювання

Область	№ ряд-ка	Всього з позитивним результатом		із них:													
				Іктеро		Помона		Грипотиф.		Гебдомад.		Канікола		Тарасов.		Інші	
		бак.	серол.	бак.	сер.	бак.	сер.	бак.	сер.	бак.	сер.	бак.	сер.	бак.	сер.	бак.	сер.
А	Б	1	2	3	4	5	6	7	8	9	10	11	12	13	14	15	16
АР Крим																	
Вінницька			15		8				1				1				5
Волинська			3		1						1		1				
Дніпропетров.		3	13	3	7												6
Донецька																	
Житомирська			5		4												1
Закарпатська			19		5		2		1		5		2				4
Запорізька																	
Івано-Франк.			20		11		5						1				3
Київська			3				2		1								
Кіровоград.			5		2						1		2				
Луганська																	
Львівська			34		5		1		7		8		1		4		8
Миколаївська			20				1				1				1		17
Одеська			2		2												
Полтавська			6		2						3						1
Рівненська			5				1		1		1						2
Сумська			7		3												4
Тернопільська		2	16		9		1		1		3		1			2	1
Харківська			3		1				1		1						
Херсонська			34		7		7		5		5		4		1		5
Хмельницька			19		5		2		3		3		4				2
Черкаська			5		3								1				1
Чернівецька			9		6				2		1						
Чернігівська			19		6		2		4		1		2		2		2
м.Київ			9		7		1		1								
м.Севастоп.																	
ЛЦ на ВТ																	
ЛЦ на ЗТ																	
ЛЦ на ПТ																	
**УКРАЇНА**		**5**	**271**	**3**	**94**		**25**		**28**		**34**		**20**		**8**	**2**	**62**

Таблиця 26. Етіологічна структура лептоспір

Особи з профілактичною метою

Область	№ ряд-ка	Всього з позитивним результатом		із них:													
				Іктеро		Помона		Грипотиф.		Гебдомад.		Канікола		Тарасов.		Інші	
		бак.	серол.	бак.	сер.	бак.	сер.	бак.	сер.	бак.	сер.	бак.	сер.	бак.	сер.	бак.	сер.
А	Б	1	2	3	4	5	6	7	8	9	10	11	12	13	14	15	16
АР Крим																	
Вінницька																	
Волинська																	
Дніпропетров.			1		1												
Донецька																	
Житомирська																	
Закарпатська			1														1

Запорізька																	
Івано-Франк.																	
Київська																	
Кіровоград.																	
Луганська																	
Львівська																	
Миколаївська																	
Одеська																	
Полтавська																	
Рівненська																	
Сумська																	
Тернопільська																	
Харківська																	
Херсонська																	
Хмельницька																	
Черкаська																	
Чернівецька																	
Чернігівська																	
м.Київ																	
м.Севастоп.																	
ЛЦ на ВТ																	
ЛЦ на ЗТ																	
ЛЦ на ПТ																	
СЕС СМСЧ																	
Крим.ПЧС																	
**УКРАЇНА**			**2**		**1**												**1**

Таблиця 26. Етіологічна структура лептоспір

Об'єкти довкілля

Область	№ ряд-ка	Всього з позитивним результатом		і з н и х :													
				Іктеро		Помона		Грипотиф.		Гебдомад.		Каніkола		Тарасов.		Інші	
		бак.	серол.	бак.	сер.	бак.	сер.	бак.	сер.	бак.	сер.	бак.	сер.	бак.	сер.	бак.	сер.
А	Б	1	2	3	4	5	6	7	8	9	10	11	12	13	14	15	16
АР Крим																	
Вінницька			2		1						1						
Волинська			44		8		3		8		15		8		1		1
Дніпропетров.			105		49		8		8				1				39
Донецька																	
Житомирська			1								1						
Закарпатська			11				2		5		1						3
Запорізька			1		1												
Івано-Франк.			19				3										16
Київська			10		4		2		2		1						1
Кіровоград.			14				5		4		1		1		2		1
Луганська																	
Львівська		1	61	1	7		31		14								9
Миколаївська			30		3				3		1		2				21
Одеська																	
Полтавська			16		3				1		1						11
Рівненська			39				10				1						28
Сумська			15		1						5				6		3
Тернопільська		17	25		1		7		2		8		6			17	1

Харківська			72		4		34		22		12						
Херсонська			73		8		15		8		3		5		10		24
Хмельницька			16						11		5						
Черкаська			3				2										1
Чернівецька			27		7		13				2						5
Чернігівська			43		11		9		5		5		2		2		9
м.Київ			15		12				3								
м.Севастоп.																	
ЛЦ на ВТ																	
ЛЦ на ЗТ																	
ЛЦ на ПТ																	
СЕС СМСЧ																	
Крим.ПЧС																	
**УКРАЇНА**		**18**	**642**	**1**	**120**		**144**		**96**		**63**		**25**		**21**	**17**	**173**

Додаток 3 до листа
ДУ "Центр громадського здоров'я
МОЗ України"
від ________ №______________

Таблиця 26. Етіологічна структура лептоспір

Хворі і особи з підозрою на захворювання

Область	№ ряд-ка	Всього з позитивним результатом		із них:													
				Іктеро		Помона		Грипотиф.		Гебдомад.		Канікола		Тарасов.		Інші	
		бак.	серол.	бак.	сер.	бак.	сер.	бак.	сер.	бак.	сер.	бак.	сер.	бак.	сер.	бак.	сер.
А	Б	1	2	3	4	5	6	7	8	9	10	11	12	13	14	15	16
АР Крим																	
Вінницька			11		8								2				1
Волинська			1								1						
Дніпропетров.			4		3												1
Донецька																	
Житомирська			1		1												
Закарпатська			22		2		7		4		7		1				1
Запорізька																	
Івано-Франк.			4		3				1								
Київська			1		1												
Кіровоград.			4		1			1					3				
Луганська																	
Львівська			5		1						1		1		1		1
Миколаївська			26				1						1				24
Одеська			5						5								
Полтавська			3		1				1								1
Рівненська			1				1										
Сумська			1														1
Тернопільська			4		2				1		1						
Харківська			1						1								
Херсонська			11		2		2		1		1		3				2
Хмельницька			13		9		1		1								2
Черкаська			3		2												1
Чернівецька			3		2						1						
Чернігівська			10		3		1		2				1				3
м.Київ			23		9		4		8				2				
м.Севастоп.																	
ЛЦ на ВТ																	
ЛЦ на ЗТ																	
ЛЦ на ПТ																	
**УКРАЇНА**			**157**		**50**		**17**	**1**	**25**		**12**		**14**		**1**		**38**

Таблиця 26. Етіологічна структура лептоспір

Особи з профілактичною метою

Область	№ ряд-ка	Всього з позитивним результатом		із них:													
				Іктеро		Помона		Грипотиф.		Гебдомад.		Канікола		Тарасов.		Інші	
		бак.	серол.	бак.	сер.	бак.	сер.	бак.	сер.	бак.	сер.	бак.	сер.	бак.	сер.	бак.	сер.
А	Б	1	2	3	4	5	6	7	8	9	10	11	12	13	14	15	16
АР Крим																	
Вінницька																	
Волинська																	
Дніпропетров.																	
Донецька																	
Житомирська																	
Закарпатська																	

Запорізька																	
Івано-Франк.																	
Київська																	
Кіровоград.																	
Луганська																	
Львівська																	
Миколаївська																	
Одеська																	
Полтавська																	
Рівненська																	
Сумська																	
Тернопільська																	
Харківська																	
Херсонська																	
Хмельницька																	
Черкаська																	
Чернівецька																	
Чернігівська																	
м.Київ																	
м.Севастоп.																	
ЛЦ на ВТ																	
ЛЦ на ЗТ																	
ЛЦ на ПТ																	
СЕС СМСЧ																	
Крим.ПЧС																	
**УКРАЇНА**																	

Таблиця 26. Етіологічна структура лептоспір

Об'єкти довкілля

Область	№ ряд-ка	Всього з позитивним результатом		і з н и х :													
				Іктеро		Помона		Грипотиф.		Гебдомад.		Каніколa		Тарасов.		Інші	
		бак.	серол.	бак.	сер.	бак.	сер.	бак.	сер.	бак.	сер.	бак.	сер.	бак.	сер.	бак.	сер.
А	Б	1	2	3	4	5	6	7	8	9	10	11	12	13	14	15	16
АР Крим																	
Вінницька																	
Волинська			16				2		1		10						3
Дніпропетров.			47		23		13		5								6
Донецька																	
Житомирська			1								1						
Закарпатська																	
Запорізька																	
Івано-Франк.																	
Київська																	
Кіровоград.																	
Луганська																	
Львівська			4						3								1
Миколаївська			10		1		3										6
Одеська																	
Полтавська																	
Рівненська																	
Сумська			1												1		

Тернопільська			3						2		1						
Харківська			4		1		1		2								
Херсонська			20		1		3		1		1		2		3		9
Хмельницька																	
Черкаська																	
Чернівецька			14		2		5		5		1						1
Чернігівська																	
м.Київ																	
м.Севастоп.																	
ЛЦ на ВТ																	
ЛЦ на ЗТ																	
ЛЦ на ПТ																	
СЕС СМСЧ																	
Крим.ПЧС																	
**УКРАЇНА**			**120**		**28**		**27**		**19**		**14**		**2**		**4**		**26**

Додаток 4 до листа
ДУ "Центр громадського здоров'я
МОЗ України"
від ________ №______________

Таблиця 26. Етіологічна структура лептоспір

Хворі і особи з підозрою на захворювання

Область	№ ряд-ка	Всього з позитивним результатом		із них:													
				Іктеро		Помона		Грипотиф.		Гебдомад.		Канікола		Тарасов.		Інші	
		бак.	серол.	бак.	сер.	бак.	сер.	бак.	сер.	бак.	сер.	бак.	сер.	бак.	сер.	бак.	сер.
А	Б	1	2	3	4	5	6	7	8	9	10	11	12	13	14	15	16
АР Крим																	
Вінницька			4		4												
Волинська			1				1										
Дніпропетров.			3														3
Донецька																	
Житомирська																	
Закарпатська			17		2				10		3						2
Запорізька																	
Івано-Франк.			18		6		7		1				3				1
Київська			2		2												
Кіровоград.			4				1						3				
Луганська																	
Львівська			11		2				1		1						7
Миколаївська			27				1										26
Одеська			2		1								1				
Полтавська			2		1				1								
Рівненська			2				1								1		
Сумська			1														1
Тернопільська			2		1						1						
Харківська																	
Херсонська			5		2								2				1
Хмельницька			3		2								1				
Черкаська			3		2		1										
Чернівецька			7		2		2				2						1
Чернігівська																	
м.Київ																	
м.Севастоп.																	
ЛЦ на ВТ																	
ЛЦ на ЗТ																	
ЛЦ на ПТ																	
**УКРАЇНА**			**114**		**27**		**14**		**13**		**7**		**10**		**1**		**42**

Додаток 1 до листа
ДУ "Центр громадського здоров'я
МОЗ України"
від ________ №______________

Таблиця 26. Етіологічна структура лептоспір

Особи з профілактичною метою

Область	№ ряд-ка	Всього з позитивним результатом		і з н и х :													
				Іктеро		Помона		Грипотиф.		Гебдомад.		Канікола		Тарасов.		Інші	
		бак.	серол.	бак.	сер.	бак.	сер.	бак.	сер.	бак.	сер.	бак.	сер.	бак.	сер.	бак.	сер.
А	Б	1	2	3	4	5	6	7	8	9	10	11	12	13	14	15	16
АР Крим																	
Вінницька																	
Волинська																	
Дніпропетров.																	
Донецька																	
Житомирська																	
Закарпатська																	
Запорізька																	
Івано-Франк.																	
Київська																	
Кіровоград.																	
Луганська																	
Львівська																	
Миколаївська																	
Одеська																	
Полтавська																	
Рівненська																	
Сумська																	
Тернопільська																	
Харківська																	
Херсонська																	
Хмельницька																	
Черкаська																	
Чернівецька																	
Чернігівська																	
м.Київ																	
м.Севастоп.																	
ЛЦ на ВТ																	
ЛЦ на ЗТ																	
ЛЦ на ПТ																	
**УКРАЇНА**																	

Таблиця 26. Етіологічна структура лептоспір

Об'єкти довкілля

Область	№ ряд-ка	Всього з позитивним результатом		і з н и х :													
				Іктеро		Помона		Грипотиф.		Гебдомад.		Канікола		Тарасов.		Інші	
		бак.	серол.	бак.	сер.	бак.	сер.	бак.	сер.	бак.	сер.	бак.	сер.	бак.	сер.	бак.	сер.
А	Б	1	2	3	4	5	6	7	8	9	10	11	12	13	14	15	16
АР Крим																	
Вінницька																	
Волинська			8				1		2		3						2

Дніпропетров.			62		9		21		6				6				20
Донецька																	
Житомирська			1								1						
Закарпатська			7				5		1								1
Запорізька																	
Івано-Франк.			8		4		4										
Київська			3		3												
Кіровоград.																	
Луганська																	
Львівська			16		2		2		12								
Миколаївська			29				1		7				5				16
Одеська																	
Полтавська			2						2								
Рівненська			28				2		5								21
Сумська																	
Тернопільська		2	19		7		4				8						
Харківська			37		3		8		8		17						1
Херсонська			24		4		2		4		3				4		7
Хмельницька																	
Черкаська																	
Чернівецька			20		3		8		4		3						2
Чернігівська																	
м.Київ																	
м.Севастоп.																	
ЛЦ на ВТ																	
ЛЦ на ЗТ																	
ЛЦ на ПТ																	
СЕС СМСЧ																	
Крим.ПЧС																	
**УКРАЇНА**		**2**	**264**		**35**		**58**		**51**		**35**		**11**		**4**		**70**

Додаток 2 до листа
ДУ "Центр громадського здоров'я
МОЗ України"
від ________ №______________

Таблиця 26. Етіологічна структура лептоспір

Хворі і особи з підозрою на захворювання

Область	№ ряд-ка	Всього з позитивним результатом		із них:													
				Іктеро		Помона		Грипотиф.		Гебдомад.		Канікола		Тарасов.		Інші	
		бак.	серол.	бак.	сер.	бак.	сер.	бак.	сер.	бак.	сер.	бак.	сер.	бак.	сер.	бак.	сер.
А	Б	1	2	3	4	5	6	7	8	9	10	11	12	13	14	15	16
АР Крим																	
Вінницька		1	5	1	5												
Волинська			2								1						1
Дніпропетров.			10		7												3
Донецька																	
Житомирська			1		1												
Закарпатська			7		2		1										4
Запорізька																	
Івано-Франк.			3		1						1						1
Київська																	
Кіровоград.																	
Луганська																	
Львівська			13		1				4						1		7
Миколаївська			1														1
Одеська																	
Полтавська			5		5												
Рівненська			2										1				1
Сумська																	
Тернопільська			3				1						2				
Харківська																	
Херсонська																	
Хмельницька			21		4		2		9		1						5
Черкаська			3						1				1				1
Чернівецька			1														1
Чернігівська																	
м.Київ																	
м.Севастоп.																	
**УКРАЇНА**		**1**	**77**	**1**	**26**		**4**		**14**		**3**		**4**		**1**		**25**

Таблиця 26. Етіологічна структура лептоспір

Особи з профілактичною метою

Область	№ ряд-ка	Всього з позитивним результатом		із них:													
				Іктеро		Помона		Грипотиф.		Гебдомад.		Канікола		Тарасов.		Інші	
		бак.	серол.	бак.	сер.	бак.	сер.	бак.	сер.	бак.	сер.	бак.	сер.	бак.	сер.	бак.	сер.
А	Б	1	2	3	4	5	6	7	8	9	10	11	12	13	14	15	16
АР Крим																	
Вінницька																	
Волинська																	
Дніпропетров.																	
Донецька																	
Житомирська																	

Закарпатська																	
Запорізька																	
Івано-Франк.																	
Київська																	
Кіровоград.																	
Луганська																	
Львівська																	
Миколаївська																	
Одеська																	
Полтавська																	
Рівненська																	
Сумська																	
Тернопільська																	
Харківська																	
Херсонська																	
Хмельницька																	
Черкаська																	
Чернівецька																	
Чернігівська																	
м.Київ																	
м.Севастоп.																	
**УКРАЇНА**																	

Таблиця 26. Етіологічна структура лептоспір

Об'єкти довкілля

**Область**	**№ ряд-ка**	**Всього з позитивним результатом**		**і з н и х :**													
				**Іктеро**		**Помона**		**Грипотиф.**		**Гебдомад.**		**Канікола**		**Тарасов.**		**Інші**	
		бак.	серол.	бак.	сер.	бак.	сер.	бак.	сер.	бак.	сер.	бак.	сер.	бак.	сер.	бак.	сер.
А	Б	1	2	3	4	5	6	7	8	9	10	11	12	13	14	15	16
АР Крим																	
Вінницька																	
Волинська			10				2		1		5				1		1
Дніпропетров.			44		22		2		5				5				10
Донецька																	
Житомирська																	
Закарпатська			1		1												
Запорізька																	
Івано-Франк.			4		3						1						
Київська																	
Кіровоград.																	
Луганська																	
Львівська			28		5		9		2		1						11
Миколаївська																	
Одеська																	
Полтавська																	
Рівненська			7						2								5
Сумська																	
Тернопільська			19		8		7		4								

Харківська																	
Херсонська																	
Хмельницька			20				3		15				2				
Черкаська			8						8								
Чернівецька			31		8		8		8		5						2
Чернігівська																	
м.Київ																	
м.Севастоп.																	
**УКРАЇНА**			**172**		**47**		**31**		**45**		**12**		**7**		**1**		**29**

Додаток 3 до листа
ДУ "Центр громадського здоров'я
МОЗ України"
від ________ №______________

Таблиця 26. Етіологічна структура лептоспір

Хворі і особи з підозрою на захворювання

Область	№ ряд-ка	Всього з позитивним результатом		із них:													
				Іктеро		Помона		Грипотиф.		Гебдомад.		Канікола		Тарасов.		Інші	
		бак.	серол.	бак.	сер.	бак.	сер.	бак.	сер.	бак.	сер.	бак.	сер.	бак.	сер.	бак.	сер.
А	Б	1	2	3	4	5	6	7	8	9	10	11	12	13	14	15	16
АР Крим																	
Вінницька			9		6												3
Волинська			4		1						3						
Дніпропетров.			35		26												9
Донецька																	
Житомирська			2		1						1						
Закарпатська			138		11		29		1		28		5				64
Запорізька																	
Івано-Франк.			31		4		8		3		12						4
Київська			2		2												
Кіровоград.			16				2		5		7						2
Луганська																	
Львівська			25		7		2		1		1				2		12
Миколаївська		20	5		2		1				1		1			20	
Одеська			6		5		1										
Полтавська			2		2												
Рівненська			9		2		2		2				3				
Сумська			2						1		1						
Тернопільська			9		3		2						2				2
Харківська			18		3		2		7		1						5
Херсонська			7		5		1						1				
Хмельницька			27		8		3		1		5		1		1		8
Черкаська			8		4				1		2						1
Чернівецька			8		6				1								1
Чернігівська			56		25				12		6		8				5
м.Київ																	
м.Севастоп.																	
**УКРАЇНА**		**20**	**419**		**123**		**53**		**35**		**68**		**21**		**3**	**20**	**116**

Таблиця 26. Етіологічна структура лептоспір

Особи з профілактичною метою

Область	№ ряд-ка	Всього з позитивним результатом		із них:													
				Іктеро		Помона		Грипотиф.		Гебдомад.		Канікола		Тарасов.		Інші	
		бак.	серол.	бак.	сер.	бак.	сер.	бак.	сер.	бак.	сер.	бак.	сер.	бак.	сер.	бак.	сер.
А	Б	1	2	3	4	5	6	7	8	9	10	11	12	13	14	15	16
АР Крим																	
Вінницька																	
Волинська																	
Дніпропетров.																	
Донецька																	
Житомирська																	

Закарпатська																	
Запорізька																	
Івано-Франк.																	
Київська																	
Кіровоград.																	
Луганська																	
Львівська																	
Миколаївська																	
Одеська																	
Полтавська																	
Рівненська																	
Сумська																	
Тернопільська																	
Харківська																	
Херсонська																	
Хмельницька																	
Черкаська																	
Чернівецька																	
Чернігівська																	
м.Київ																	
м.Севастоп.																	
**УКРАЇНА**																	

Таблиця 26. Етіологічна структура лептоспір

Об'єкти довкілля

**Область**	**№ ряд-ка**	**Всього з позитивним результатом**		**і з н и х :**													
				**Іктеро**		**Помона**		**Грипотиф.**		**Гебдомад.**		**Канікола**		**Тарасов.**		**Інші**	
		бак.	серол.	бак.	сер.	бак.	сер.	бак.	сер.	бак.	сер.	бак.	сер.	бак.	сер.	бак.	сер.
А	Б	1	2	3	4	5	6	7	8	9	10	11	12	13	14	15	16
АР Крим																	
Вінницька																	
Волинська			15				3		3		7				1		1
Дніпропетров.			169		87		5						29				48
Донецька																	
Житомирська			3		2						1						
Закарпатська			16				13				1						2
Запорізька																	
Івано-Франк.			13		1		4		4		1		1				2
Київська			4		1				1				2				
Кіровоград.																	
Луганська																	
Львівська			22		4		1		12								5
Миколаївська		2	1										1			2	
Одеська			10		2		4								1		3
Полтавська																	
Рівненська			21												3		18
Сумська			3		1		1										1
Тернопільська			22		4		3		5				3				7

Харківська			17		5		4		5		2						1
Херсонська			50		17		17						9				7
Хмельницька			8								8						
Черкаська																	
Чернівецька			33		6		8		10		3						6
Чернігівська			135		37		12		19		15		21		18		13
м.Київ																	
м.Севастоп.																	
**УКРАЇНА**		**2**	**542**		**167**		**75**		**59**		**38**		**66**		**23**	**2**	**114**

Table S1. Panel of Leptospira Reference Strains Used in the Microscopic Agglutination Test, According to the Respective Serovars and Serogroups (Methodological Recommendations 2002)

№	Serogroup	Serovar	Reference strain
1	Grippotyphosa	Grippotyphosa	Moskva V
2	Icterohaemorrhagiae	Copenhageni	M-20
3	Canicola	Canicola	Hond-Utrecht IV
4	Pomona	Pomona	Pomona
5	Tarassovi	Tarassovi	Perepelicin
6	Hebdomadis	Cabura	Cabura
7	Sejroe[a]	Polonica wolffi	493 Poland 3705
8	Javanica	Poi	Poi
9	Autumnalis	Autumnalis	Akijami A
10	Australis	Bratislava	Jez Bratislava
11	Bataviae	Djatzi	HS-26
12	Ballum	Ballum	Muz-127
13	Pyrogenes	Pyrogenes	Salinen
14	Cynopteri	Cynopteri	Vleermuis 3868

Official Response on Leptospirosis Incidence and Serogroup Prevalence in Ukraine

These letters provide an official response from the Regional CDC and the Ukrainian Public Health Centre regarding the incidence of leptospirosis and the prevalence of serogroups in humans and animals across Ukraine and its regions.
